# Dynamic Tensor Modeling for Missing Data Completion in Electronic Toll Collection Gantry Systems

**DOI:** 10.3390/s24010086

**Published:** 2023-12-23

**Authors:** Yikang Rui, Yan Zhao, Wenqi Lu, Can Wang

**Affiliations:** 1 School of Transportation, Southeast University, Nanjing 211189, China; 101012189@seu.edu.cn (Y.R.);; 2 Joint Research Institute on Internet of Mobility, Southeast University and University of Wisconsin-Madison, Southeast University, Nanjing 211189, China

**Keywords:** tensor Tucker decomposition, dynamic tensor modeling, data imputation, data integrity

## Abstract

The deployment of Electronic Toll Collection (ETC) gantry systems marks a transformative advancement in the journey toward an interconnected and intelligent highway traffic infrastructure. The integration of these systems signifies a leap forward in streamlining toll collection and minimizing environmental impact through decreased idle times. To solve the problems of missing sensor data in an ETC gantry system with large volumes and insufficient traffic detection among ETC gantries, this study constructs a high-order tensor model based on the analysis of the high-dimensional, sparse, large-volume, and heterogeneous characteristics of ETC gantry data. In addition, a missing data completion method for the ETC gantry data is proposed based on an improved dynamic tensor flow model. This study approximates the decomposition of neighboring tensor blocks in the high-order tensor model of the ETC gantry data based on tensor Tucker decomposition and the Laplacian matrix. This method captures the correlations among space, time, and user information in the ETC gantry data. Case studies demonstrate that our method enhances ETC gantry data quality across various rates of missing data while also reducing computational complexity. For instance, at a less than 5% missing data rate, our approach reduced the RMSE for time vehicle distance by 0.0051, for traffic volume by 0.0056, and for interval speed by 0.0049 compared to the MATRIX method. These improvements not only indicate a potential for more precise traffic data analysis but also add value to the application of ETC systems and contribute to theoretical and practical advancements in the field.

## 1. Introduction

Electronic Toll Collection (ETC) systems signify a remarkable advancement in vehicular management, enabling swift identification and seamless toll transactions. ETC gantry data are characterized by their large volume, diverse modalities, rapid acquisition, coexistence of genuine and erroneous data, and rich potential for transportation applications. These systems are primarily classified into two categories: toll plazas equipped with dedicated ETC lanes and overpass gantries [1,2]. Notably, China has taken a pioneering role in this technological transformation since 2019, systematically phasing out provincial boundary toll stations in favor of the widespread deployment of ETC gantries [3]. This bold initiative has led to the establishment of a robust network, supported by over 230 million ETC users, marking the dawn of a new era in toll collection [4].

ETC systems represent a significant technological advancement in traffic management, characterized by high-quality, extensive coverage and the widespread accessibility of data. These data, renowned for their comprehensiveness, play a crucial role in enabling precise traffic analyses [5,6,7]. However, operating such advanced systems comes with a unique set of challenges, primarily influenced by factors such as system equipment performance, the installation of image automatic capture and recognition systems, weather and lighting conditions, and the proper functioning of equipment [8].

These factors often lead to common issues within ETC gantry system, such as unrecorded license plates, misidentifications, and delayed data uploads [9]. Unrecorded license plates can result from various factors, like poor visibility, suboptimal camera angles, or technical glitches in recognition software, significantly impacting the accuracy and reliability of the data, which, in turn, affect toll collection and traffic monitoring processes. Misidentification of license plates often occurs because of limitations in the optical character recognition technology, especially under challenging conditions, such as low light, adverse weather, or high vehicle speeds. Such errors can lead to incorrect toll charges, causing inconvenience to users and operational complexities for the authorities. Additionally, delayed data uploads pose a significant challenge in systems designed for real-time or near-real-time processing. Delays in data transmission can disrupt the efficient functioning of traffic management systems, especially in situations in which immediate data availability is critical for decision making, like in traffic congestion management or emergency response scenarios. Another noteworthy challenge is the significant variation in distances between ETC gantries, which creates substantial obstacles for seamless traffic monitoring. This irregular spacing can lead to inconsistencies in data collection, with shorter distances potentially resulting in redundant data capture and longer distances risking the omission of important traffic pattern changes.

The complexities and challenges inherent in ETC gantry systems have become pivotal areas for research and innovation. Addressing these challenges necessitates the development of sophisticated solutions that can effectively manage these complexities and enhance the overall efficacy of ETC systems. Our study aims to contribute to this field by introducing a novel approach that not only addresses the technical challenges but also significantly improves the operational aspects of ETC systems, ensuring more accurate and reliable traffic management.

We introduce high-order tensor modeling methods for ETC hub data, as well as ETC hub data completion methods based on improved tensor decomposition techniques. The structure of this article is as follows: We briefly summarize the research results of previous researchers in Section 2. In Section 3, we introduce the high-order tensor model we adopted, including the high-order tensor model for ETC hub data and the data completion method based on improved tensor decomposition technology. In particular, a detailed introduction to high-order tensor models for ETC hub data is provided in Section 3.1, including subtensor block models and extensions of tensor block models. Section 3.2 explains the ETC hub data completion method based on improved tensor decomposition techniques, including a dynamic tensor decomposition model and a sparse control Laplace matrix. Section 4 provides our experimental results and analysis, including the preprocessing of ETC license plate data, the accuracy evaluation, and the computational complexity evaluation. Section 5 summarizes the main findings and contributions of this study and discusses future research directions.

## 2. Related Works

A comprehensive analysis of prior research will enable us to identify the challenges and gaps that this study seeks to address, making an exploration of related work essential. Facing these challenges, missing data completion has emerged as a vital research frontier. Established methods encompass statistical, deep learning, and tensor-based approaches. On the basis of statistical methods, regression analysis methods using techniques such as linear regression, polynomial regression, and regression trees, are suitable for situations in which there are few missing data but that do not capture the complex relationships among data [10,11,12]. Clustering methods can group the samples in a dataset and then use other samples within the same cluster to estimate the missing values, but clustering methods need to pre-specify the number of clusters, which is limited for high-dimensional data analysis [13]. Methods based on moment decomposition include Singular Value Decomposition (SVD) and Nonnegative Matrix Factorization (NMF), which are widely used in recommender systems and image processing. However, SVD and NMF may be sensitive to outliers, may not work well when dealing with sparse matrices, and are usually used for numerical data, not for categorical data [14]. Bayesian methods allow for the introduction of uncertainty into the estimation of missing values and provide estimates of probability distributions. Markov Chain Monte Carlo (MCMC) and Bayesian networks are part of the Bayesian approach. However, in the case of high-dimensional and large-scale data, the computational complexity of Bayesian methods increases and requires a priori knowledge to specify the probability distribution [15]. Deep learning techniques, such as Recurrent Neural Networks (RNNs) and transformers, have made significant progress in data completion tasks. They can handle complex data patterns and long-term dependencies. However, complex machine learning models may require more computational resources and a large amount of labeled data for training, which, in some cases, may not be easily accessible [16,17,18,19,20]. For tensor-based complementation methods applied to high-dimensional tensors, the increase in dimensionality may lead to dimensionality catastrophe, i.e., the data become very sparse, increasing the probability of missing data points, increasing the difficulty in the complementation problem, leading to an increase in uncertainty in the results of the complementation, and the model suffers from overfitting or difficulty in generalizing to new data [21,22,23].

The intricacy of traffic flow data, with its multifaceted correlations and patterns, necessitates analytical approaches that can adeptly manage their complexity. Tensor models have risen to prominence in this context, offering a robust framework for capturing high-order correlations and multimodal data interactions inherent in traffic systems [24,25]. These models are particularly valued for their ability to exploit the intrinsic low-rank properties of traffic data, thus enhancing the precision and interpretability of the analyses. A significant contribution in this domain has been made by Chen et al., who utilized Gaussian regular polynomial decomposition to unravel the latent structures within the data, applying Bayesian networks to fill in the gaps of incomplete datasets [15]. Their work stands out for integrating probabilistic models with tensor decomposition, providing a powerful tool for data restoration and uncertainty quantification in traffic flow analysis. Expanding upon these foundational models, subsequent research has delved into dynamic tensor flow models that aim to capture the spatiotemporal dynamics of traffic flow. These models are sophisticated, as they are designed to analyze data across both macroscales, which consider long-term trends and cyclic patterns, and microscales, which focus on the minute-by-minute fluctuations of traffic flow [26,27,28]. By doing so, they provide a granular view of traffic dynamics, offering insight into the temporal progression and spatial distribution of traffic congestion, vehicle speeds, and density. However, the application of these models is not without challenges. One critical issue is the computational and storage inefficiencies that arise from the redundant calculations within tensor windows, which often result in increased processing time and memory requirements [29]. This redundancy is particularly problematic when dealing with large-scale traffic datasets, where the efficiency of computation can significantly impact the timeliness and usability of the analysis.

To address these inefficiencies, there has been a push toward optimizing tensor calculations, such as employing more sophisticated tensor decomposition techniques that reduce redundancy and accelerate computation. These techniques include the use of block term decomposition, which partition the tensor into smaller, more manageable blocks, and the implementation of parallel computing strategies that distribute the workload across multiple processors [30]. Furthermore, advancements in hardware, such as the use of GPUs for tensor operations, have also contributed to alleviating the computational burden [30]. In summary, while existing tensor models have laid a solid foundation for traffic data analysis, there is a continual need for innovation to overcome the computational challenges associated with these complex models. Our research contributes to this field by proposing a novel tensor model that not only captures the high-order correlations and spatiotemporal dynamics of traffic flow but also improves computational efficiency through optimized decomposition techniques and algorithmic enhancements.

To combat the aforementioned deficiencies, particularly with large volumes of ETC gantry data, our study introduces an innovative method for missing data completion that leverages an enhanced dynamic tensor decomposition technique. This method not only acknowledges the high-dimensional nature of ETC gantry data but also pioneers the use of a high-order tensor model, refined through an improved tensor Tucker decomposition process. The main contributions of our study are manifold: (1) A dynamic tensor flow model was proposed for decomposing the high-order tensor model of ETC gantry data after analyzing the characteristics of ETC gantry data. (2) The issue of high sparsity in ETC gantry data was addressed through the introduction of an adaptive tensor window to analyze incremental tensor blocks in dynamic tensor flow. Furthermore, an improvement was made to the incremental processing method of the dynamic tensor flow by incorporating a Laplacian matrix. (3) An approximate computation approach was adopted to handle the decomposition of neighboring tensor blocks within the same group, leveraging the characteristics of the gantry data, leading to the computational speed enhancement of the high-order tensor Tucker decomposition.

## 3. Materials and Methods

### 3.1. High-Order Tensor Model for ETC Gantry Data

**Figure 1:**
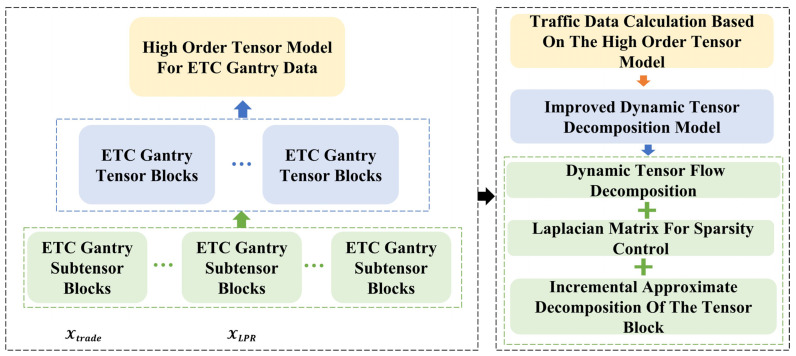
Traffic information extraction process for ETC gantry data.

ETC gantry data on highways exhibit multifaceted characteristics, including complex structural patterns, rapid update frequencies, and varied density values across different data types. To address these complexities, this section introduces a high-order tensor model framework specifically designed for ETC gantry data. This framework aims to filter out attributes that do not pertain to traffic analysis and to streamline the dimensionality of the data. The extraction methodology is depicted in Figure 1.

In Figure 1, we provide an overview of our proposed high-order tensor model for processing ETC gantry data. The figure illustrates the architecture of the model, which consists of two main components: the high-order tensor model for ETC gantry data on the left and the traffic data calculation process based on the model on the right. The left component encapsulates ETC gantry data in the form of tensor blocks, indicating the multidimensional properties of data collected from various gantry structures. This is further decomposed into subtensor blocks for detailed analysis and processing. The right component shows the sequence of our improved dynamic tensor decomposition model, which starts from a high-order tensor model and is decomposed through dynamic tensor flow decomposition, combined with the Laplace matrix for sparsity control, and reaches its climax through the incremental approximate decomposition of the tensor blocks. The interaction between the subtensor blocks and improved dynamic decomposition model is the core of achieving precise traffic data computation, which is the ultimate goal of our research.

#### 3.1.1. Multidimensional Tensor Construction for ETC Gantry Data Analysis

**Figure 2:**
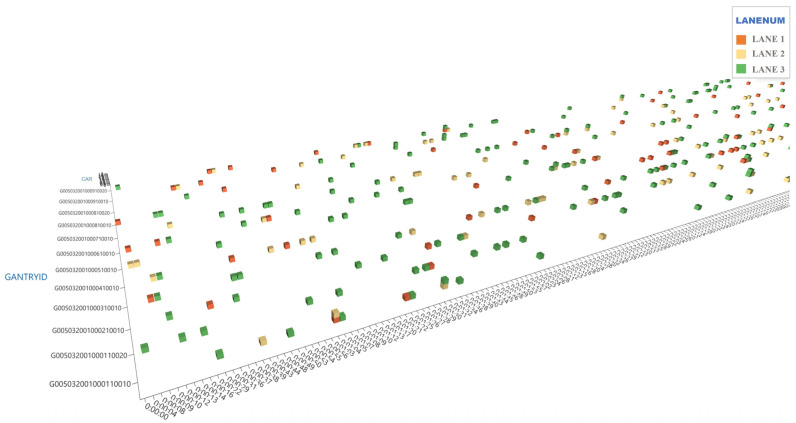
Diagram of the three-dimensional tensor model of ETC gantry data.

We construct a three-dimensional tensor model that intricately integrates time, gantry space, and vehicle user dimensions. We measure the time dimension in seconds, setting the minimum counting unit at one second, and tailoring the parameters to reflect the dynamics of traffic flow accurately. The statistical duration determines the time dimension’s granularity. The gantry space dimension captures the driving direction, gantry number, and functionality, providing a spatial context for our analysis. We define the vehicle user dimension through parameters such as license plate information, vehicle model, and transaction status, ensuring a comprehensive categorization. Our tensor model meticulously records vehicle passage times, offering granular insight into the temporal flow, while the spatial dimension is marked by specific gantry identifiers. However, the model’s expansion across time, space, and vehicle diversity leads to increased computational complexity and tensor size—a challenge compounded by significant sparsity. Large periods and areas lacking vehicle transactions result in an unnecessarily inflated tensor, complicating data storage and computation. A three-dimensional tensor model of the ETC gantry data is shown in Figure 2.

Figure 2 visually represents the three-dimensional tensor model for ETC gantry data. In this model, the *x*-axis represents time, segmented into units of 4 s each, capturing the temporal aspect of traffic flow. The *y*-axis corresponds to the gantry ID, delineating the specific location of each gantry along the highway. The *z*-axis indicates the presence or absence of vehicles in different lanes at any given time. Distinct colors represent different lanes for ease of interpretation: orange for the first lane, yellow for the second, and green for the third. This color-coding aids in visualizing the distribution and movement of traffic across various lanes. The diagram exemplifies the model’s capacity to encapsulate detailed information about vehicle flow, including temporal and spatial dynamics. However, it also highlights the challenge of tensor inflation due to large periods and areas lacking vehicle transactions, which can complicate data storage and computational processes.

To address these issues, our analysis employs innovative techniques to compress the tensor effectively. These methods enable us to discern significant patterns within the sparse dataset, preserving the fidelity of the temporal and spatial details. Our approach ensures that the model remains both manageable and reflective of the intricate patterns within traffic data.

#### 3.1.2. ETC Gantry Tensor Block and Subtensor Block Models

This study introduces gantry tensor block models to encapsulate the traffic statistics of a gantry over one-minute intervals. These models facilitate streamlined calculations through a methodically organized sequence. In alignment with high-order tensor models, these blocks adopt time, space, and vehicle user dimensions as their fundamental structure. Each time dimension within a tensor block corresponds to 1 min, comprising 60 distinct time components. The spatial dimension is bifurcated into 2 key factors: gantry number and gantry malfunction. The vehicle user dimension mirrors that of the high-order tensor model, ensuring consistency. Crucially, the process of dimensionality reduction applied to ETC gantry data retains the intrinsic properties of the original high-dimensional dataset within a more manageable, low-dimensional framework. For practical implementation, attributes such as ETC gantry number, capture time, and license plate identification were meticulously selected to construct a subtensor block model, as follows:

(1) $$X_{LPR}\in R^{{\;I}_{time}\times I_{space}\times I_{car}}\;I_{time}=\left[t_{pictime}\right]\;I_{space}=\left[s_{id}\right]\;\;\;I_{car}=\left[c_{license}\right]$$

Herein, $X_{LPR}$ represents the subtensor block of the ETC gantry plate recognition data, encompassing the capture time, $I_{time}=\left[t_{pictime}\right]$; gantry number,$I_{space}=\left[s_{id}\right]$; and identified license plate, $I_{car}=\left[c_{license}\right]$.

Similarly, the ETC gantry transaction data are synthesized into a subtensor block model, effectively enabling the assessment of transactional patterns:

(2) $$X_{trade}\in R^{\;I_{time}\times I_{space}\times I_{car}}\;I_{time}=\left[t_{tradetime}\right]\;I_{space}=\left[s_{id}\right]\;\;\;\;I_{car}=\left[c_{license},c_{type},c_{match}\right]$$

Herein, $X_{trade}$ represents the subtensor block of the ETC gantry transaction data, encompassing the capture time, $I_{time}=\left[t_{tradetime}\right]$; gantry number, $I_{space}=\left[s_{id}\right]$; and vehicle license plate recognition information, vehicle type, and transaction outcome, $I_{car}=\left[c_{license},c_{type},c_{match}\right]$.

#### 3.1.3. Extension of the Subtensor Block Models to the Tensor Block Model

The subtensor block models are integrated into a higher-order tensor space; components within the same dimension and of identical order are merged, preserving the unique orders. The amalgamation of subtensor blocks is executed as follows:

(3) $$A\in R^{I\times J\times K}\;I=\left[i_{1},i_{2},i_{3}\right],\;J=\left[j_{1},j_{2},j_{3}\right],K=\left[k_{1},k_{2}\right]\;B\in R^{I\times J},I=\left[i_{1},i_{2},i_{4}\right],J=\left[j_{1},j_{2}\right]\;f:A\overset{⃑}{\times }B\to C,\;C\in R^{I\times J\times K}\;\;\;\;I=\left[i_{1},i_{2},i_{3},i_{4}\right],J=\left[j_{1},j_{2},j_{3}\right],K=\left[k_{1},k_{2}\right]$$

Equation (3) delineates the method for merging subtensor blocks. In this context, $A\in R^{I\times J\times K}$ and $B\in R^{I\times J}$ represent tensor blocks with different dimensions, where $A\in R^{I\times J\times K}$ has tensor dimensions $I=\left[i_{1},i_{2},i_{3}\right],J=\left[j_{1},j_{2},j_{3}\right],\;and\;K=\left[k_{1},k_{2}\right]$, and $B\in R^{I\times J}$ has tensor dimensions $I=\left[i_{1},i_{2},i_{4}\right]\;and\;J=\left[j_{1},j_{2}\right]$. $A\in R^{I\times J\times K}$ and $B\in R^{I\times J}$ are superimposed to form tensor $C\in R^{I\times J\times K}$ with dimensions $I=\left[i_{1},i_{2},i_{3},i_{4}\right],J=\left[j_{1},j_{2},j_{3}\right],\;and\;K=\left[k_{1},k_{2}\right]$.

By expanding each subtensor block model, we construct a tensor block model, which is then sequentially organized along the timeline to form a composite, higher-order tensor model. Parameters of identical order within each dimension constitute the foundational attribute parts, while those of varying orders are categorized as extensible attribute parts, facilitating a modular approach to model construction.

#### 3.1.4. Traffic Data Calculation Based on the High-Order Tensor Model

**Figure 3:**
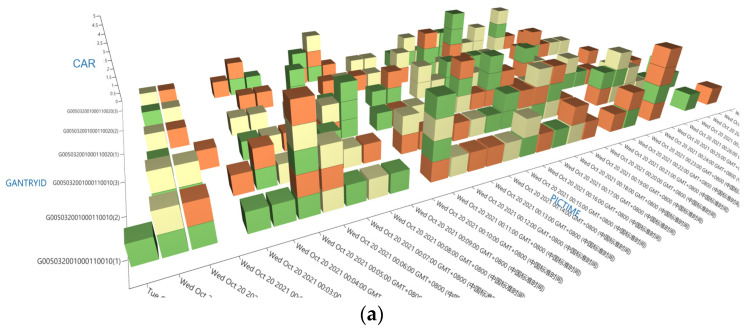

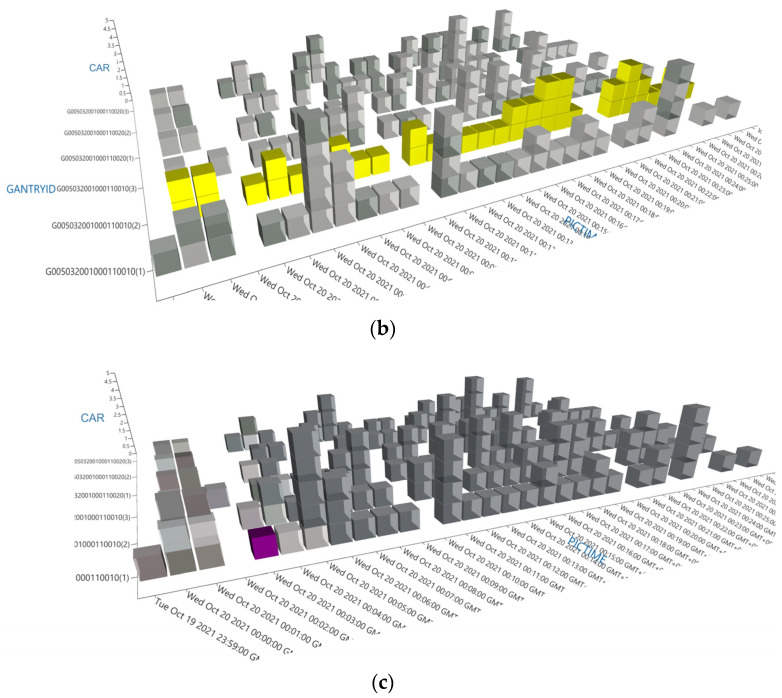
Diagrammatic representations of traffic data calculations based on the high-order tensor model: (**a**) layout of high-order tensor model; (**b**) dissection of the high-order tensor model to analyze traffic data (**c**) process of extracting specific vehicle information within the high-order tensor mode for traffic.

Traffic data extraction was performed through calculations using a high-order tensor model. Figure 3a depicts this model, which utilizes 30 min traffic statistics from two ETC gantries, each with three lanes. In this model, the vehicle types within tensor blocks are color-coded, enhancing the model’s visual interpretation. This tensor model is adaptable to various spatial component transformations—including expansion, reflection, shear, projection, and rotation—enabling complex, multidimensional traffic data calculations.

For instance, Figure 3b illustrates the calculation of traffic data for lane 2 at the G005032001000110010 gantry. By segmenting a slice of the tensor model for this specific gantry, the model yields comprehensive traffic information for lane 2. This slice encompasses tensor blocks representing different time intervals and crucial traffic parameters, such as traffic volume, average time headway, vehicle type distribution, and space occupancy rate. Additionally, the process of retrieving vehicle-level information is demonstrated in Figure 3c. By entering the license plate details of a specific vehicle, the model directly locates the corresponding tensor block. Subsequently, traffic parameters, including interval speed and time headway for that vehicle, are accurately computed.

### 3.2. ETC Gantry Data Completion Method Based on Improved Tensor Decomposition

This study introduces an Improved Dynamic Tensor Decomposition (IDTD) model, specifically designed to extract high-dimensional and dynamic traffic features with enhanced accuracy and efficiency. To counter the sparsity issue prevalent in high-dimensional data, a Laplacian matrix was employed to regulate parameter sparsity. Furthermore, approximate calculations were conducted to ascertain the tensor core and factor matrices, significantly reducing computational overhead.

#### 3.2.1. Improved Dynamic Tensor Decomposition Model

The sparse nature of traffic data presents significant challenges. The “vehicle user-time stamp” information matrix typically exhibits low density, which becomes increasingly problematic as the volume of highway vehicles and the frequency of traffic record timestamps surge, thereby diminishing the information density related to vehicles. The addition of a spatial dimension exacerbates this sparsity. Our approach involves analyzing tensor flow within a stipulated timeframe and introducing a tensor window to the data tensor flow. The tensor flow for a fixed period with a tensor window size of w is denoted as:

(4) $$\begin{array}{r} D\left(t,w\right)=\left\{X_{\left(T-w+1\right)},\ldots ,X_{T}\right\}\\ X_{t}\in R^{I_{1}\times I_{2}\times ,...,I_{N+1}} \end{array}$$

**Figure 4:**
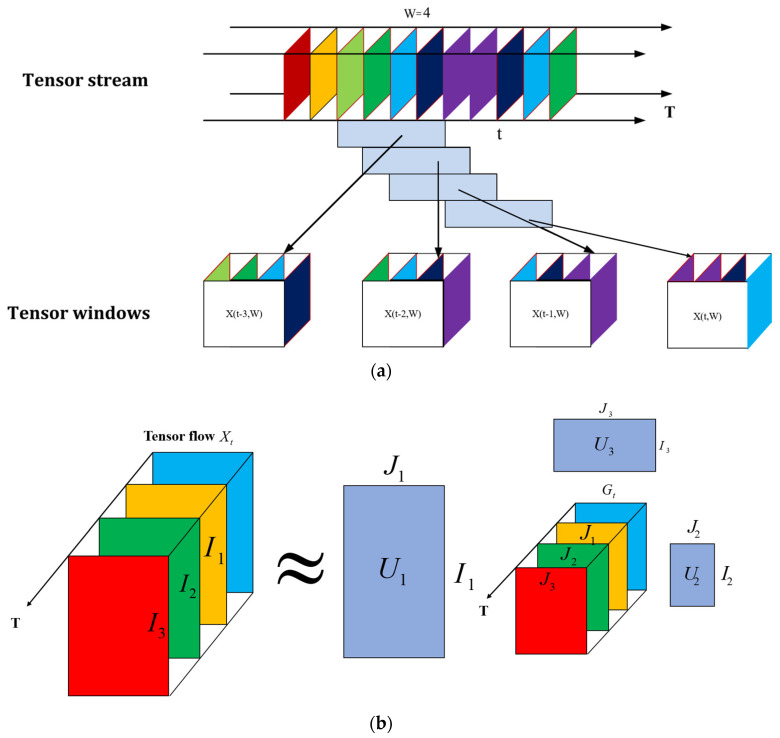
Diagrams of the dynamic tensor flow decomposition: (**a**) tensor window diagram; (**b**) diagram of the tensor flow decomposition.

As depicted in Figure 4a, the tensor flow $X_{t}\in R^{I_{1}\times I_{2}\times ,...,I_{N}},\left(1\leq t\leq T\right)$ performs local processing using a sliding tensor window of size w. Moreover, dynamic traffic flow information is captured using the dynamic tensor model. The decomposition of the Nth-order tensor flow $X_{t}\in R^{I_{1}\times I_{2}\times ,...,I_{N}},\left(1\leq t\leq T\right)$, is articulated as:

(5) $$X_{t}\approx G_{t}\times_{1}U_{1}\times_{2}U_{2}\times \ldots \ldots \times_{N}U_{N}+E_{t}\;\;\;G_{t}\in R^{J_{1}\times J_{2},...,\times J_{N}}$$

where $U_{n}$ represents the factor matrix corresponding to mode n (the principal component of mode n); $G_{t}$ is the core tensor, encapsulating independent features that articulate the interactions among various pattern principal components; and $U_{1},U_{2},{and\;U}_{3}$ are the factor matrices for modes 1, 2, and 3, respectively. The constituents of the core tensor stream,$G_{t}$, mirror the interplay between principal components and the temporal dynamics of this tensor series. The intricacies of tensor flow decomposition are exemplified in Figure 4b.

#### 3.2.2. Laplacian Matrix for Sparsity Control

**Figure 5:**
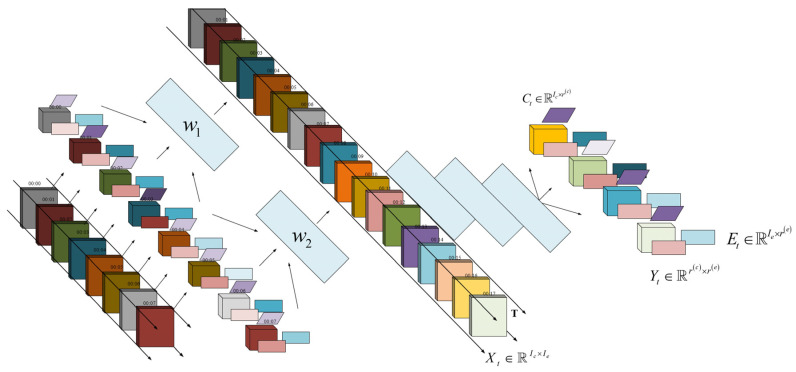
Dynamic tensor processing of ETC gantry traffic data.

The dynamic tensor flow decomposition model for ETC gantry data is illustrated in Figure 5. This model postulates that a segment of vehicle passage information is represented by the tuple $\left(c,e,v,t\right)$, where the passage information v for vehicle c at gantry e is recorded in period t,(t=1,...,T). The ETC gantry data, structured as a time-series, form a dynamic tensor sequence $X_{t}\in R^{n_{c}\times n_{e}\times n_{v}}$, where $n_{c}$ is the count of vehicles passing within the chosen time, $n_{e}$ denotes the quantity of ETC gantries, and $n_{v}$ represents the volume of passage data points. Leveraging Tucker decomposition, the dynamic tensor sequence decomposes as:

(6) $$X_{t}\approx Y_{t}\times_{\left(c\right)}C_{t}\times_{\left(e\right)}E_{t}\times_{\left(v\right)}V_{t}$$

where $Y_{t}\in R^{r^{\left(c\right)}\times r^{\left(e\right)}\times r^{\left(v\right)}}$ is the core tensor sequence encapsulating dynamic behavioral patterns that describe the interplay among vehicles, gantries, and passage information. It signifies the likelihood of the occurrence for passage information from group $j^{\left(v\right)}$ at group $j^{\left(e\right)}$ gantries for vehicles belonging to group $j^{\left(c\right)}$ before time t.

The matrix $C_{t}\in R^{n_{c}\times r^{\left(c\right)}}$ serves as the vehicle factor matrix up until time t, where $C_{t}\left(i^{\left(c\right)},j^{\left(c\right)}\right)$ indicates the probability that the $i^{\left(c\right)}th$ vehicle belongs to the $j^{\left(c\right)}th$ vehicle group.

The matrix $E_{t}\in R^{n_{e}\times r^{\left(e\right)}}$ is the factor matrix for the ETC gantries before time t, and $E_{t}\left(i^{\left(e\right)},j^{\left(e\right)}\right)$ illustrates the likelihood that the $i^{\left(e\right)}th$ gantry belongs to the $j^{\left(e\right)}th$ gantry group.

The matrix $V_{t}\in R^{n_{v}\times r^{\left(v\right)}}$ represents the factor matrix for passage information until time t, and $V_{t}\left(i^{\left(v\right)},j^{\left(v\right)}\right)$ denotes the probability that the $i^{\left(v\right)}th$ passage belongs to the $j^{\left(v\right)}th$ passage information group.

The tensor model for ETC gantry traffic data is inherently intricate. While large tensor windows yield strong classification capabilities, they concomitantly escalate computational complexity [31]. To maintain the precision of tensor approximation decomposition within homogenous tensor groups, we introduced variable tensor window sizes. The interrelation among discrete tensor blocks was quantified using the Pearson correlation coefficient $r_{ij}$, setting the tensor window size predicated on the threshold $r_{ij}>0.95$. The Pearson correlation coefficient and the corresponding tensor window size are computed as follows:

(7) $$r_{ij}=\frac{COV\left(X_{i},X_{j}\right)}{\sqrt{\sigma_{X_{i}}\sigma_{X_{j}}}}$$

(8) $$w_{i}=\sum_{j=1}^{T}\mathrm{sgn}(r_{ij})\mathrm{sgn}(r_{ij})=\left\{\begin{array}{c} 1,\;r_{ij}\geq 0.95\\ 0,\;r_{ij}<0.95 \end{array}\right.$$

**Figure 6:**
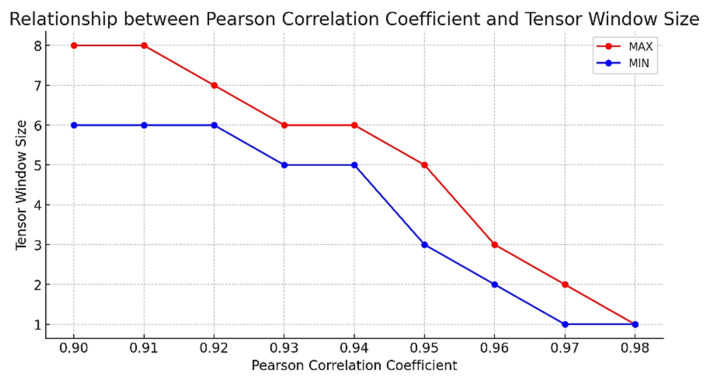
Maximum and minimum tensor window values based on varying criteria.

Figure 6 illustrates the range of maximum and minimum tensor window values, which vary according to different correlation coefficients. To minimize the computational load while preserving the integrity of the tensor decomposition approximations, this study synthesized the established discriminative threshold of the Pearson correlation coefficient with the concrete context of the ETC gantry data [32]. A correlation coefficient exceeding 0.95 $\left(r_{ij}>0.95\right)\;is$ indicative of a strong correlation, prompting the adjustment of the tensor window size to this stringent standard.

To address the challenges posed by high data sparsity, the Laplacian matrices $L_{\left(c\right)},L_{\left(e\right)},\;and\;L_{\left(v\right)}$ were employed, quantifying the similarity among individual entities and their collective groups, namely, passing vehicles, ETC gantries, and transit information. The Laplacian matrix is defined as:

(9) L=D−W

where D is the degree matrix, and W is the similarity matrix. The $\left(i,j\right)th$ matrix element represents the similarity between the ith and jth entities. The similarity degrees for vehicles, gantries, and transit data are tailored according to parameters like car model, geographic proximity, and interval headway time. Direct comparisons among entities are facilitated by numerical differentials, with vehicle model specifics calibrated by the conversion factor detailed in Table 1.

The elements of the similarity matrix, W, along each column are aggregated to yield n values, and these n values are then positioned on the main diagonal to construct the degree matrix, D, as an n×n diagonal matrix, leaving the nondiagonal entries at zero. The derived Laplacian matrices, $L_{\left(c\right)},L_{\left(e\right)},\;and\;L_{\left(v\right)}$, are symmetric and positive semi-definite, each with its minimum eigenvalue being zero. This characteristic is instrumental for the execution of incremental approximate decomposition of the tensor block, simplifying the computational process involved in the analysis of the ETC gantry data.

#### 3.2.3. Calculation of the Incremental Approximate Decomposition of the Tensor Block

The ETC gantry data exhibit a substantial spatial correlation. Given the minor variations within neighboring tensor blocks, these correlations can be harnessed to conserve computational resources. The sparse increment at time t, denoted by $\Delta X_{t}=X_{t+1}-X_{t}$, facilitates a more efficient dynamic tensor decomposition.

1. Initialization of the Core Tensor and Constraint Terms:

Utilizing Tucker decomposition, we acquire the initial tensor $X_{1}$, alongside the constraint term $L_{\left(m\right)}{|}_{m=1}^{M}\in R^{n_{m}\times n_{m}}$, within the M-dimensional tensor sequence $X_{t}\in R^{I_{1}\times I_{2}\times ...\times I_{m}}$.

2. Initial Tensor Covariance Matrix Calculation:

At time t=1, the covariance matrix for the mth dimension of the initial tensor $X_{1}$ is defined as:

(10) $${C_{1}}^{m}=X_{1}^{\left(m\right)}X_{1}^{\left(m\right)T}+\mu_{\left(m\right)}L_{\left(m\right)}$$

where ${X_{1}}^{\left(m\right)}$ is the unfolded matrix of $X_{1}$ along the mth dimension, and $\mu_{\left(m\right)}$ is the weight for $L_{\left(m\right)}{|}_{m=1}^{M}\in R^{n_{m}\times n_{m}}$.

3. Factor Matrix and Core Tensor Calculation:

The factor matrix $U_{1}^{\left(m\right)}\in R^{n_{m}\times r_{m}}$ comprises the leading $r_{m}$ eigenvectors of the covariance matrix, ${C_{1}^{m}|}_{m=1}^{M}$. The core tensor $Y_{1}\in R^{r_{1}\times \ldots \times r_{M}}$ is computed correspondingly. This procedure is outlined in Table 2.

4. Update of the Factor Matrix $U_{t+1}^{\left(m\right)}$

The revised factor matrix, $U_{t+1}^{\left(m\right)}$, is realized through the calculation of the update of the eigenvalue, $\lambda_{t+1,i}^{m}$, and the eigenvector, $u_{t+1,i}^{m}$, as ${C_{t}}^{m}=X_{t}^{\left(m\right)}X_{t}^{\left(m\right)T}+\mu_{\left(m\right)}L_{\left(m\right)}$, whose eigenvalue is $\lambda_{t,i}^{m}$ with an eigenvector of $u_{t,i}^{m}$; the size of the change in $X_{t}$ is $\Delta X_{t}$.

(11) $$\lambda_{t+1,i}^{m}=\lambda_{t,i}^{m}+\Delta \lambda_{t,i}^{m}$$

(12) $$u_{t+1,i}^{m}=u_{t,i}^{m}+\Delta u_{t,i}^{m}$$

Bringing the expressions for $\lambda_{t+1,i}^{m}$ and $u_{t+1,i}^{m}$ into ${C_{t+1}}^{m}$ and omitting t simplifies the equation.

(13) $$\left[(X^{\left(m\right)}+\Delta X^{\left(m\right)})(X^{\left(m\right)}+\Delta X^{\left(m\right)}{)}^{T}+\mu_{\left(m\right)}L_{\left(m\right)}](u_{i}^{m}+\Delta u_{i}^{m}\right)\;\;=(\lambda_{i}^{m}+\Delta \lambda_{i}^{m})(u_{i}^{m}+\Delta u_{i}^{m})$$

By focusing only on first-order variable terms, we simplify to:

(14) $$\begin{array}{ll} X^{\left(m\right)}X^{\left(m\right)T} & \Delta u_{i}^{m}+(X^{\left(m\right)}\Delta X^{\left(m\right)T}+\Delta X^{\left(m\right)}X^{\left(m\right)T})u_{i}^{m}+\mu_{\left(m\right)}L_{\left(m\right)}\Delta u_{i}^{m}\\ & =\lambda_{i}^{m}\Delta u_{i}^{m}+\Delta \lambda_{i}^{m}u_{i}^{m} \end{array}$$

The orthogonality of eigenvectors permits the characterization of the changes in $\Delta u_{i}^{m}$ using the current eigenvectors:

(15) $$\Delta u_{i}^{m}\approx \sum_{j=1}^{r_{m}}\alpha_{ij}u_{j}^{m}$$

where $\left\{\alpha_{ij}\right\}$ is the constant to be calculated and substituted to obtain:

(16) $$\begin{array}{ll} (X^{\left(m\right)}X^{\left(m\right)T} & +\mu_{\left(m\right)}L_{\left(m\right)})\sum_{j=1}^{r_{m}}\alpha_{ij}u_{j}^{m}+\left(X^{\left(m\right)}\Delta X^{\left(m\right)T}+\Delta X^{\left(m\right)}X^{\left(m\right)T}\right)u_{i}^{m}\\ & =\lambda_{i}^{m}\sum_{j=1}^{r_{m}}\alpha_{ij}u_{j}^{m}+\Delta \lambda_{i}^{m}u_{i}^{m} \end{array}$$

The above equation can be further simplified by multiplying the left-hand side of the equation by ${\left(u_{k}^{m}\right)}^{T}$, and the above equation can be further simplified:

(17) $$\lambda_{k}^{m}\alpha_{ik}+{\left(u_{k}^{m}\right)}^{T}(X^{\left(m\right)}\Delta X^{\left(m\right)T}+\Delta X^{\left(m\right)}X^{\left(m\right)T})u_{i}^{m}\;=\lambda_{i}^{m}\alpha_{ik}$$

(18) $$\alpha_{ik}=\frac{{\left(u_{k}^{m}\right)}^{T}(X^{\left(m\right)}\Delta X^{\left(m\right)T}+\Delta X^{\left(m\right)}X^{\left(m\right)T})u_{i}^{m}}{\lambda_{i}^{m}-\lambda_{k}^{m}}$$

The eigenvectors satisfy orthogonal unitization, which yields the following:

(19) $$(u_{i}^{m}+\Delta u_{i}^{m}{)}^{T}(u_{i}^{m}+\Delta u_{i}^{m})=1\Leftrightarrow 1+2(u_{i}^{m}{)}^{T}\Delta u_{i}^{m}+ο(||\Delta u_{i}^{m}|{|}^{2})=1$$

Eliminating the higher-order regression term leads to $\alpha_{ii}=0$.

(20) $$\Delta u_{i}^{m}=\sum_{j\neq i}\frac{{\left(u_{j}^{m}\right)}^{T}(X^{\left(m\right)}\Delta X^{\left(m\right)T}+\Delta X^{\left(m\right)}X^{\left(m\right)T})u_{i}^{m}}{\lambda_{i}^{m}-\lambda_{j}^{m}}u_{j}^{m}$$

5. Updating the Core Tensor, $Y_{t+1}\in R^{r_{1}\times ...\times r_{M}}$

The core tensor can be updated according to the updating of the factor matrix, $U_{t+1}^{\left(m\right)}\in R^{n_{m}\times r_{m}}$, and the calculation process for the core tensor $Y_{t+1}\in R^{r_{1}\times ...\times r_{M}}$ is summarized in Table 3.

#### 3.2.4. ETC Gantry Data Completion Based on Improved Tensor Dynamic Decomposition

Restoration of missing ETC gantry data leverages the correlation among inter-tensor block traffic data. We approximate the kernel tensor for missing data by analyzing the decomposition of surrounding tensor blocks.

#### 3.2.5. Description of the ETC Gantry Data Completion Issue

It is assumed that there exists a higher-order tensor block, $X\in R^{I_{1}\times I_{2}\times I_{3}}$, with missing values and a set of observable data within X, which is $\Phi \in [I_{1}]\times [I_{2}]\times [I_{3}]$. Given that $x_{ijk}\left(i,j,k\right)\in \Phi$, the elemental values outside the observable dataset should be calculated as rapidly as possible while ensuring accuracy. Therefore, the observable dataset within X is projected onto each spatial dimension of the tensor block as follows: if (i,j,k)∈Φ, then the observed value of element $P_{\Phi }(x)$ is $x_{ijk}$; otherwise, the elemental value is zero.

(21) $${Ρ}_{\Phi }X=\left\{\begin{array}{cc} x_{ijk}, & \left(i,j,k\right)\in \Phi \\ 0, & \mathrm{else} \end{array}\right.$$

where $P_{\Phi }$ is the projection operator.

#### 3.2.6. Construction of the Objective Function

The method adopted for ETC gantry data completion, rooted in Tucker decomposition, addresses the associated optimization problem.

(22) $$f\left(G,U^{\left(1\right)},U^{\left(2\right)},U^{\left(3\right)}\right)=\mathrm{argmin}\frac{1}{2}{\left\|{Ρ}_{\Phi }-G\times_{1}U^{\left(1\right)}\times_{2}U^{\left(2\right)}\times_{3}U^{\left(3\right)}\right\|}_{2}^{2}$$

To minimize the function, the core tensor G and factor matrices $U^{\left(1\right)},U^{\left(2\right)},\;and\;U^{\left(3\right)}$ are the factor matrices to be completed. The current function can be minimized by obtaining the core tensor and factor matrix.

#### 3.2.7. Solution to the Objective Function

1. Initial Solution:

Taking into account the properties of adjacent tensor blocks, the initial solution for the objective function is estimated using the tensor core and factor matrices, represented as $G_{0},U_{0}^{\left(1\right)},U_{0}^{\left(2\right)},\;and\;U_{0}^{\left(3\right)}$.

2. Optimization of Solutions:

The solution is refined through iterative computation of the approximate gradients for each variable. This iterative process ensures the optimal update of the objective function, guided by the following update rule:

(23) $$\begin{array}{ll} G_{k}= & \mathrm{argmin}\left\langle \nabla_{G}f\left(G_{k-1},\tilde{U}\right),G-G_{k-1}\right\rangle +\frac{L_{G}}{2}{\left\|G-G_{k-1}\right\|}_{F}^{2}+\lambda_{i}{\left\|G\right\|}_{1}\\ & \;\;\;=\max\left|0,G_{k-1}-\frac{1}{L_{G}}\nabla_{G}f(G_{k-1},\tilde{U})-\frac{\lambda_{G}}{L_{G}}\right| \end{array}$$

(24) $$\begin{array}{ll} U_{k}^{\left(i\right)}= & \mathrm{argmin}\left\langle \nabla_{U^{\left(i\right)}}f(\tilde{G},{\tilde{U}}^{\left(j\neq i\right)},U^{\left(i\right)}),U-U_{k-1}^{\left(i\right)}\right\rangle +\frac{L_{U^{\left(i\right)}}}{2}{\left\|U-U_{k-1}^{\left(i\right)}\right\|}_{F}^{2}\\ & \;\;\;+\lambda_{i}{\left\|U^{\left(i\right)}\right\|}_{1}=\max\left|0,U_{k-1}^{\left(i\right)}-\frac{1}{L_{U^{\left(i\right)}}}\nabla_{U^{\left(i\right)}}f(\tilde{G},{\tilde{U}}^{\left(j\neq i\right)},U^{\left(i\right)})-\frac{\lambda_{i}}{L_{i}}\right| \end{array}$$

In the above equations, $\nabla_{G}f\left(G_{k-1},\tilde{U}\right)\;and\;\nabla_{U^{\left(i\right)}}f(\tilde{G},{\tilde{U}}^{\left(j\neq i\right)},U^{\left(i\right)})$ are the gradients of $f(G,U^{\left(1\right)},U^{\left(2\right)},U^{\left(3\right)})$ to G and $U^{\left(i\right)}$, respectively, and $L_{G}$ and $L_{U^{\left(i\right)}}$ are the Lipschitz constants of the corresponding gradient function, respectively, satisfying the Lipschitz conditions.

(25) $$\left\|f\left(G_{k-1}\left(1\right),\tilde{U}\right)-f\left(G_{k-1}\left(2\right),\tilde{U}\right)\right\|\leq L_{G}{\left\|G\left(1\right)-G\left(2\right)\right\|}_{F},\forall G\left(1\right),G\left(2\right)$$

(26) $$\left\|f\left(\tilde{G},{\tilde{U}}^{\left(j\neq i\right)},U_{k-1}^{\left(i1\right)}\right)-f\left(\tilde{G},{\tilde{U}}^{\left(j\neq i\right)},U_{k-1}^{\left(i2\right)}\right)\right\|\leq L_{U^{\left(i\right)}}{\left\|U^{\left(i1\right)}-U^{\left(i2\right)}\right\|}_{F},\forall U^{\left(i1\right)},U^{\left(i2\right)}$$

The flow of the ETC gantry data completion calculation, based on tensor decomposition, is presented in Table 4.

## 4. Results

### 4.1. ETC Gantry Plate Data Preprocessing

Our empirical analysis centered on a segment of the Shanghai–Chongqing Expressway, specifically the G50 Nanxun to Jingze Interchange (G50 Nanjing section). Over the 30 days from 1–30 January 2022, we collated 24 sets of ETC gantry data via the toll system on this stretch. These datasets comprise both static information (including vehicle, toll station, and pass card details, alongside toll system specifics) and dynamic records (detailing travel, toll transactions, and system validations). This study focused on the investigation and analysis of ETC gantry plate recognition data for a specific road section. Three main types of anomalies in ETC gantry plate recognition data were identified: incorrect plate recognition data, missing plate recognition data, and duplicate plate recognition data. The causes and forms of these anomalies are outlined in Table 5:

Directly applying abnormal ETC gantry plate recognition data in the computation of traffic parameters can severely disrupt model results and analyses. The preprocessing of abnormal plate recognition data in this section includes the identification of ETC gantry abnormal plate recognition data based on classification concepts, preliminary repair of abnormal plate recognition data, and evaluation of the integrity of ETC gantry plate recognition data.

Initially, by establishing a normal plate recognition data model using standards and norms, one can predetermine the characteristics of normal data within the data table. This includes correct data formats/rules and distribution models for each attribute. The ETC gantry plate recognition data table is then checked against these standards, including data format, degree of fit within distribution models, repetition of vehicle passage information through each gantry, and vehicle travel direction. Differences between the tested data and the normal data characteristics are used to identify anomalies.

In the second step, the prematch of abnormal ETC gantry plate recognition data involves the correction of erroneous, duplicate, and missing data through deletion, expansion, completion, and transformation, thus enhancing the data usability. As indicated above, different processing methods can be selected based on the cause and form of the anomalies. For incorrect plate recognition data, identified errors are deleted from the standard data table and then reclassified as missing data, moving into the repair process for missing plate recognition data. The issue of missing data is resolved using automated completion and repair methods based on auxiliary analysis data tables, ETC gantry toll transaction tables, and ETC gantry abnormal event logs, which are manually processed, to ensure the accuracy of the data and assist in the completion of missing ETC gantry plate recognition data. For instance, if part of the data for a vehicle are missing in the gantry plate recognition data table, one can use the complete information available for that vehicle within the table to search the entire vehicle passage record in the auxiliary analysis tables, utilizing the correlation of the same vehicle’s short-term operational status to extract usable data for completion. In cases where multiple identical entries appear in the ETC gantry plate recognition data table, the uniqueness of the billing transaction identification number is used as a standard to retain one instance of the duplicate plate recognition data and discard the rest.

Finally, this paper quantitatively describes errors, omissions, and duplications in plate recognition data information and evaluates the quality of ETC gantry plate recognition data. A commonly used data quality evaluation index formula is as follows:

(27) $${TD}_{IN}=\frac{{NUM}_{valid}}{{NUM}_{all}}$$

Within the formula, ${NUM}_{valid}$ represents the amount of valid data in the original ETC gantry plate recognition data table that is nonredundant and complete. ${NUM}_{all}$ is the theoretical total amount of data that should be in the ETC gantry plate recognition data table. ${TD}_{IN}$ is the evaluation index for the quality of the ETC gantry plate recognition data.

Issues such as network transmission delays can lead to delayed uploads of plate recognition data. The ETC gantry system tolerates delays in data upload to a certain extent, but uploads exceeding a specific time range can affect highway toll transactions. Therefore, this section sets t as the acceptable time range. When the data upload time is within this range, it is marked as a normal upload; otherwise, it is considered delayed. The data evaluation index formula is adjusted as follows:

(28) $${TD}_{IN}=\frac{{NUM}_{obtain}-{NUM}_{duplicate}-{NUM}_{delay}}{{NUM}_{all}}$$

**Figure 7:**
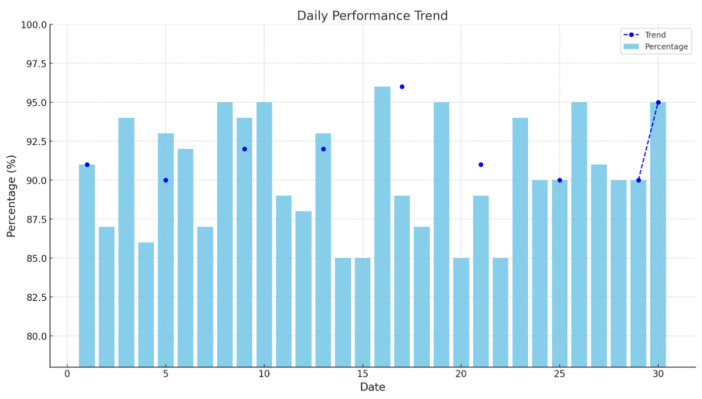
The data quality of the G50 Nanjing section’s ETC gantry for January.

Here, ${NUM}_{delay}$ represents the volume of delayed uploaded data. ${NUM}_{duplicate}$ is the volume of duplicate data in the ETC gantry plate recognition data table. ${NUM}_{obtain}$ is the actual total volume of data obtained by the ETC gantry. After identifying and initially repairing the abnormal data, the quality of the ETC gantry plate recognition data is evaluated. The data quality of the G50 Nanjing section ETC gantry for January is illustrated in Figure 7.

**Figure 8:**
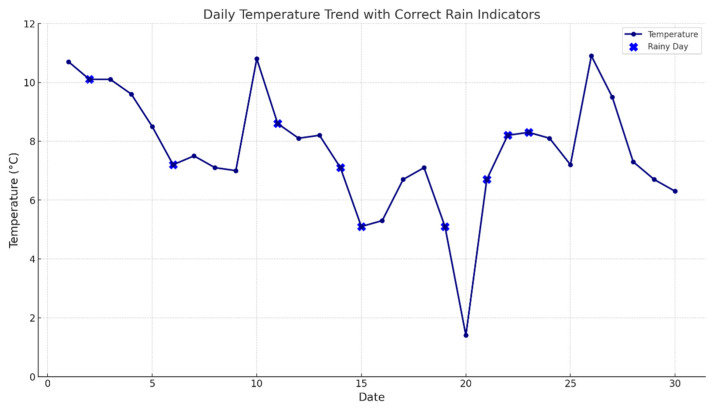
Meteorological conditions in the Suzhou Area of the G50 Nanjing Section.

The quality of the ETC gantry data was generally high, with the monthly quality level exceeding 85% and an average data quality level of 90.4% for the month. There were 11 days in the month when the ETC gantry data quality fell below 90% (referred to as poor data quality), which is already superior to the detection effects of most detectors on highways. Utilizing meteorological data from the ETC gantry system’s built-in weather detection equipment and referencing weather data, an investigation was conducted on the meteorological conditions in January 2022 in the area of the G50 Nanjing section (Suzhou). The region experienced minimal variation in January temperatures, with an average temperature of 7.93 °C, including an outlier of 1.4 °C. The region’s precipitation for the month was 74.8 mm over 9 days of rainfall, with the specific weather conditions shown in Figure 8 below.

Given the minor fluctuations in regional temperatures and its location in the south, where there are no cold temperatures, it can be inferred that temperature has a minimal impact on the ETC gantry’s detection ability. This paper primarily analyzed the quality of ETC gantry data under poor visibility conditions, such as overcast and rainy days. By comparing the meteorological conditions on dates when the ETC gantry data quality was above 90% with those below 90%, it was found that the proportion of rainy days with poor quality was 77.78% of the total number of rainy days and 63.64% of the total number of days with poor quality. This suggests that the quality of the ETC gantry data is affected by adverse weather conditions such as rainfall.

The accuracy and reliability of the ETC plate recognition data directly impact the operation of highway toll collection. An analysis of the actual usage of the G50 Nanjing section’s ETC gantry data reveals the following advantages: (1) Quick updates, with a large volume of data accumulating over a short period of time. Millions of vehicle passage data can be generated daily. Moreover, the data have high consistency, strong structure, and low update costs. (2) Rich dimensions of ETC gantry data provide multiple perspectives for traffic data analysis. The ETC gantry system includes static data, such as vehicle information, toll station information, pass card information, and toll system information, as well as dynamic data, like travel information, charging information, and system verification information.

**Figure 9:**
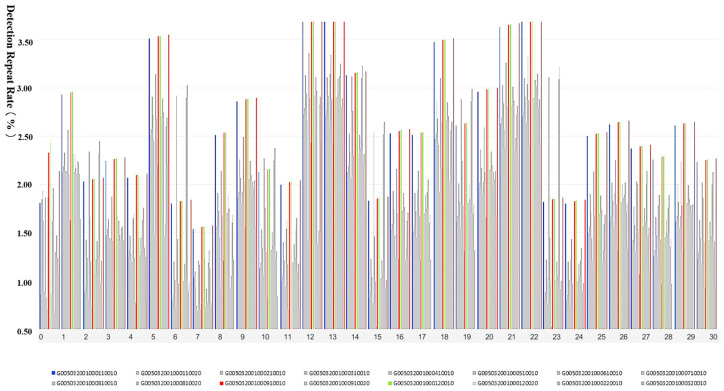
Proportion of duplicate detection data within ETC gantry segments.

The collection, transmission, and storage of plate recognition data require a multitude of devices and network services, which introduces the possibility of missed captures and identification errors: (1) Evasion tactics such as covering license plates, along with unstable equipment conditions, can result in missing fields and data anomalies within ETC gantry data, making it impossible to guarantee data quality. Concurrently, improper use by drivers, such as excessive speed, loss of ETC electronic tags, the application of special windshield films, and small vehicles being obscured by larger vehicles in tow, can lead to detection failures at the gantry, impacting data accuracy and leading to issues such as transaction anomalies, nonrecognition of vehicles, and missing vehicle statistics in the G50 Nanjing section ETC gantry. (2) The ETC gantry span in the G50 Nanjing section reaches up to 9 km, with insufficient monitoring of traffic flow between gantries. Additionally, the uneven and sparse distribution of ETC gantry detectors may lead to biases in the description of vehicle behavior. For instance, a vehicle with a license plate ending in 375 recorded passage times at two gantries, G005032001000310010 and G005032001000410010, approximately 2 min apart. With the gantries about 6 km apart, this would calculate to a speed of 180 km/h, which is inconsistent with road traffic rules. (3) ETC gantry video detectors are used for vehicle plate data collection. These video detectors rely on optical principles, and factors such as dust, shadows, weather, and lighting can affect the detection results; hence, natural elements like rainy weather and nighttime conditions impact the accuracy of gantry detection. (4) The minimum distance between gantries in the same direction on the G50 Nanjing section is set at 400 m, with two ETC gantries spaced 500 m apart, and three road segments have relatively small distances between them. Figure 9 below shows the proportion of duplicate vehicle detection data for January among ETC gantry segments, with blue, red, and green representing the three closely spaced segments, and grey representing other segments. Duplicate detection issues are more significant in the three closely spaced segments.

For tensor model construction, PyTorch 1.11 was the framework of choice. The subtensor block models were formulated using data fields from each ETC gantry within the G50 Nanjing section, encompassing aspects such as token recognition, transactions, foundational information, and atypical state indicators. These subtensor components were methodically categorized per dimension into basic and expandable attributes. The expandable attributes were then amalgamated and preserved based on the foundational attributes of each dimension to establish a three-dimensional, high-order tensor block model for the ETC gantry system.

The method’s efficacy was gauged by its accuracy and computational complexity. We contrasted this approach with a matrix decomposition-based completion model (MATRIX) and a prediction model founded on Long Short-Term Memory (LSTM) neural networks. The MATRIX model relies on the low-rank nature of matrices and known elements to decompose the target matrix into two lower-rank matrices, thereby reconstructing the missing entries [33]. Conversely, the LSTM-based model capitalizes on the predictive capabilities of neural networks for ETC gantry data restoration. Preliminary processing of the ETC gantry data revealed notable instances of missing information for January, which included discrepancies arising from incorrect data removal and delays in data transmission. These instances are encapsulated in Table 6.

In each missing data situation, two days of missing data were selected as the original data input, and the average time headway, ${h_{t}}^{i,j}$; traffic volume, $q_{i}$; and mean interval speed, ${\bar{v_{s}}}^{\left(i,i+n\right)}$ were the traffic data to be completed. After the data are calibrated using the transaction data sheet, the data can be considered accurate and set to the true value. The Root Mean Square Error (RMSE) was selected as the evaluation index for this data supplementation, and the equation is as follows:

(29) $$RMSE=\sqrt{\frac{\sum_{i=1}^{n}(\hat{x_{i}}-x_{i}{)}^{2}}{n}}$$

where $\hat{x_{i}}$ is the value of complementary traffic data, $x_{i}$ is the true value of traffic data, and n is the number of complementary traffic data points.

### 4.2. Accuracy Evaluation

**Figure 10:**
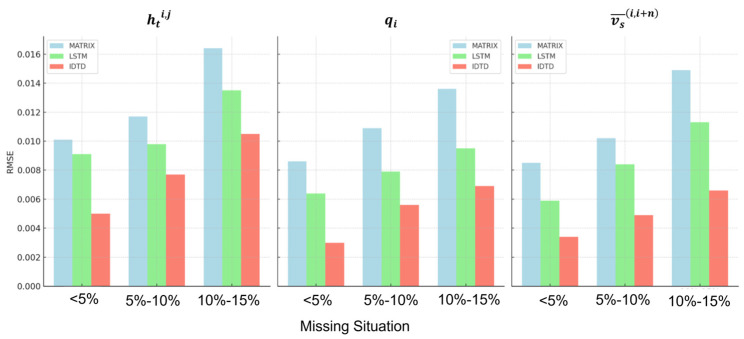
Traffic data completion comparison under different missing data scenarios.

The traffic data completion error values for the MATRIX, LSTM, and IDTD models under various missing data scenarios are presented in Table 7, with a comparative analysis depicted in Figure 10.

On the basis of the above results, it can be seen that IDTD outperformed the other two algorithms for all types of traffic data completion under different missing data situations:

- All three algorithms increased the completion error with an increase in the missing rate, among which MATRIX had the greatest effect on the completion with the missing rate, and IDTD has the least effect with the missing rate and could better adapt to severe missing data;
- Among the selected traffic data, the time headway completion error was the largest, whereas the traffic volume and interval average speed completion errors were similar. The time headway was influenced by the driving environment and independent choices of the driver. It was less effective for the low-dimensional MATRIX and only useful for the time-series LSTM.

### 4.3. Evaluation of the Computational Complexity

The evaluation of the computational complexity in this study focused on the static tensor calculation model used for real-time update passage records into the historical data and global data calculations. For our ETC gantry tensor model, the number of gantries is denoted as m, the number of vehicles recorded historically is n, the number of updated vehicles is Δn, and the number of core tensor and factor matrix iterations in the static high-order ETC gantry tensor model is $ο(m^{2}(n+\Delta n{)}^{2})$. The daily traffic volume on the G50 Nanjing highway is more than 20,000 vehicles; therefore, the calculation volume under the static tensor model is extremely large. If $D^{\left(i\right)}$ is the number of features of each point in the ith-dimensional space in the improved dynamic tensor decomposition model for ETC gantries, the high sparsity of the ETC gantry data leads to $D^{\left(i\right)}\ll \prod n^{i}$. Thus, the computational complexity of the factor matrix increment in each dimension in the conventional calculation is $ο(n^{\left(i\right)}D^{\left(i\right)})$, whereas the computational complexity of the factor matrix increment in the approximate tensor decomposition is only $ο(D^{\left(i\right)})$. The computational time complexity for updating the core tensor corresponding to the static high-order tensor decomposition and IDTD are $ο(T\sum_{i=1}^{3}\left(D^{\left(i\right)}(n^{\left(i\right)}{)}^{2}+(n^{\left(i\right)}{)}^{3}\right))$ and $ο(T\sum_{i=1}^{3}r^{\left(i\right)}(n^{\left(i\right)}+1)D^{\left(i\right)})$, respectively, which show that the computational complexity is significantly reduced.

### 4.4. Discussion

This paper’s analysis indicates that the abnormal phenomenon of the ETC gantry data is not only accidental but also indicates systematic problems from technical failures to deliberate avoidance strategies. We identified and corrected data anomalies based on the completion model. The initial repair strategy, including deletion, expansion, completion, and transformation, greatly improved the availability of data, ensuring that the subsequent analysis was based on as accurate data as possible.

The impact of weather and other environmental conditions on data quality cannot be ignored. The research results of G50 indicate that adverse weather conditions, especially rainfall, can have a negative impact on the quality of ETC gantry data. This is a key area of future research, as developing more powerful systems that can maintain high data quality regardless of environmental conditions is crucial for the reliability of traffic data analysis.

The IDTD model was used for tensor decomposition and compared with the MATRIX and LSTM models, demonstrating the potential of advanced computing techniques to address the challenges of data sparsity and complexity. The excellent performance of the IDTD model in accuracy and reducing computational load indicates its potential applicability in a range of scenarios where large-scale sparse datasets are prevalent. This study lays the foundation for future research. It is necessary to further improve data preprocessing techniques, develop more flexible data collection systems for environmental factors, and explore the scalability of IDTD models.

## 5. Conclusions

This paper introduced a high-order tensor model tailored for ETC gantry data that adeptly captures temporal and spatial structural information. Through the utilization of an Improved Dynamic Tensor Decomposition (IDTD) approach, we successfully addressed the challenges posed by the considerable sparsity of ETC gantry data. By employing a Laplacian matrix within the IDTD framework, we effectively mitigated computational burdens and preserved essential traffic characteristics in the data completion process. Our comparative analysis, illustrated through a case study, confirms the superior accuracy and reduced computational complexity of the IDTD-based algorithm over traditional methods. The IDTD method, therefore, holds substantial promise for comprehensive ETC gantry data completion and has the potential for broader application across diverse datasets and scenarios. Future research will explore extending the reach of IDTD in handling large-scale ETC gantry datasets and its adaptability to other data-intensive domains.

## Figures and Tables

**Table 1:** Numerical conversion factors for vehicle types.

Vehicle Type	Number
Minibus	*1.0*
LargeBus	*1.5*
Minivan	*2.0*
Medium-sized truck	*2.5*
Large truck	*4.0*
Extra-large truck	*5.0*

**Table 2:** Initial core tensor and factor matrix calculation process.

**Input:** $X_{1}$, $L_{\left(m\right)}{|}_{m=1}^{M}\in R^{n_{m}\times n_{m}}$ **Output:** $Y_{1},\;{{\;U}_{1}^{m}|}_{m=1}^{M},\;{\lambda {\;}_{1}^{m}|}_{m=1}^{M}$	
**Begin**	
**1**	for m=1:M
**2**	$C_{1}^{m}u_{1,i}^{m}=\lambda_{1,i}^{m}u_{1,i}^{m}$ ## Calculate the first $r_{m}$ eigenvalues, $\lambda_{1,i}^{m}$, with eigenvector $u_{1,i}^{m}$ of $C_{1}^{m}.$
**3**	$U_{1}^{\left(m\right)}=diag(u_{1,1}^{m},\ldots ,u_{1,m}^{m}$)
**4**	end for
**5**	Return $Y_{1},\;{{\;U}_{1}^{m}|}_{m=1}^{M},\;{\lambda {\;}_{1}^{m}|}_{m=1}^{M}$
**End**	

**Table 3:** Calculation flow for updating the core tensor.

**Input:** $X_{t},\;{\Delta X}_{t},\;U_{t}^{\left(m\right)},\lambda_{t}^{m}\;$ **Output:** $Y_{t+1},U_{t+1}^{\left(m\right)}{|}_{m=1}^{M},\lambda_{t+1}^{m}{|}_{m=1}^{M}$	
**Begin**	
**1**	for m=1:M
**2**	for $i=1:{\;r}^{m}$
**3**	$\lambda_{t+1,i}^{m}=\lambda_{t,i}^{m}+{\Delta \lambda }_{t,i}^{m}$ ## update the factor matrix
**4**	$u_{t+1,i}^{m}=u_{t,i}^{m}+{\Delta u}_{t,i}^{m}$
**5**	$U_{t+1}^{\left(m\right)}=diag(u_{t+1,1}^{m},\ldots ,u_{t+1,m}^{m}$)
**6**	end for
**7**	end for
**8**	$Y_{t+1}=(X_{t}+{\Delta X}_{t})\prod_{m=1}^{M}\times_{\left(m\right)}U_{t+1}^{\left(m\right)T}$
**9**	Return $Y_{t+1},U_{t+1}^{\left(m\right)}{|}_{m=1}^{M},\lambda_{t+1}^{m}{|}_{m=1}^{M}$
**End**	

**Table 4:** Computational process for ETC gantry data completion via tensor decomposition.

Input: $X\in R^{I_{1}\times I_{2}\times I_{3}}$, Dynamic Tensor Flow $W_{T}$, Iteration error $\varepsilon_{0}=10^{-6}$ Output: $X_{NEW}$	
**Begin**	
**1**	Calculate ${Ρ}_{\Phi }X$;
**2**	Apply $W_{T}$ approximate tensor decomposition to obtain the initial solution $G_{0},\;U_{0}^{\left(1\right)},\;U_{0}^{\left(2\right)},\;U_{0}^{\left(3\right)}$; ## initialization of the objective function solution
**3**	Calculate $\varepsilon =\frac{{\left\|X-X_{NEW}\right\|}_{F}}{{\left\|X\right\|}_{F}}$; ## Error calculation
**4**	While $\varepsilon >\varepsilon_{0}$, do ## Set iteration conditions
**5**	$G_{k}=\max\left|0,G_{k-1}-\frac{1}{L_{G}}\nabla_{G}f(G_{k-1},\tilde{U})-\frac{\lambda_{G}}{L_{G}}\right|$;
**6**	$U_{k}^{\left(i\right)}=\max\left|0,U_{k-1}^{\left(i\right)}-\frac{1}{L_{U^{\left(i\right)}}}\nabla_{U^{\left(i\right)}}f(\tilde{G},{\tilde{U}}^{\left(j\neq i\right)}U^{\left(i\right)})-\frac{\lambda_{i}}{L_{i}}\right|$;
**7**	end for
**8**	$X_{NEW}=G_{k}\times_{1}U_{k}^{\left(1\right)}\times_{2}U_{k}^{\left(2\right)}\times_{3}U_{k}^{\left(3\right)}$
**9**	Return $X_{NEW}=G_{k}\times_{1}U_{k}^{\left(1\right)}\times_{2}U_{k}^{\left(2\right)}\times_{3}U_{k}^{\left(3\right)}$
**End**	

**Table 5:** Causes and forms of anomalies in ETC gantry plate recognition data.

	Causes	Forms
**Incorrect Plate Recognition**	Detector malfunction	Unrecognized characters (e.g., “A00000_9” as a default)
	Upload failure or nonstandard storage	Vehicle plate numbers with fewer digits than normal, as well as other data format errors; sudden changes in driving parameters, vehicle plate numbers with fewer digits than normal
	Vehicle evasion	Parts of the plate not existing in reality, indicative of toll evasion
**Missing Plate Recognition**	High traffic flow parameters, weather, operator error, and detector failure High traffic flow parameters, weather, operator error	Absence of vehicle passage information in the data table; Absence of vehicle passage information
	Communication network failures, power supply issues	Complete or partial absence of vehicle passage information; Complete or partial absence of vehicle passage information
**Duplicate Plate Recognition**	Vehicle plate data transmission fault	A single vehicle detected by an ETC gantry within a short interval (typically within 10 s)
	Adjacent detectors duplicate detection (i.e., interference between gantries)	Multiple ETC gantries recording the passage information of the same vehicle within a short time frame (typically within 10 s)

**Table 6:** Overview of missing ETC gantry data in January.

Missing Data	<5%	5–10%	10–15%
Number of days	7	12	11

**Table 7:** RMSE values for traffic data completion.

Missing Situation	<5%	5–10%	10–15%	<5%	5–10%	10–15%	<5%	5–10%	10–15%
	MATRIX			LSTM			IDTD		
${h_{t}}^{i,j}$	0.0101	0.0117	0.0164	0.0091	0.0098	0.0135	0.0050	0.0077	0.0105
$q_{i}$	0.0086	0.0109	0.0136	0.0064	0.0079	0.0095	0.0030	0.0056	0.0069
${\bar{v_{s}}}^{\left(i,i+n\right)}$	0.0085	0.0102	0.0149	0.0059	0.0084	0.0113	0.0034	0.0049	0.0066

## Data Availability

Data are contained within the article.

## References

[1] Randriamasy M., Cabani A., Chafouk H., Fremont G. (2019). Geolocation process to perform the electronic toll collection using the ITS-G5 technology. IEEE Trans. Veh. Technol..

[2] Lin M.Y., Chen Y.C., Lin D.Y., Hwang B.F., Hsu H.T., Cheng Y.H., Liu Y.T., Tsai P.J. (2020). Effect of implementing electronic toll collection in reducing highway particulate matter pollution. Environ. Sci. Technol..

[3] Hong Y., Sha Y., Ding F., Chen Z., Zhang D. (2023). Resource allocation optimization and performance evaluation for 5G cellular vehicle-to-everything (C-V2X). J. Automot. Saf. Energy.

[4] Chen H., An B., Sharon P., Miao C., Soh Y. (2018). DyETC: Dynamic Electronic Toll Collection for Traffic Congestion Alleviation. Thirty-Second. AAAI Conf. Artif. Intell. (AAAI-18).

[5] Chen Z., Wu B., Li B., Ruan H. (2021). Expressway exit traffic flow prediction for ETC and MTC charging system based on entry traffic flows and LSTM model. IEEE Access.

[6] Zhao Y., Lu W., Rui Y., Ran B. (2023). Classification of the Traffic Status Subcategory with ETC Gantry Data: An Improved Support Tensor Machine Approach. J. Adv. Transp..

[7] Wang J., Zhu R., Li T., Gao F., Wang Q., Xiao Q. (2021). ETC-oriented efficient and secure blockchain: Credit-based mechanism and evidence framework for vehicle management. IEEE Trans. Veh. Technol..

[8] Ding F., Mi G., Tong E., Zhang N., Bao J., Zhang G. (2022). Multi-channel high-resolution network and attention mechanism fusion for vehicle detection model. J. Automot. Saf. Energy.

[9] Zhao Y., Zhang B., Cao Y., Rui Y., Ran B. (2022). Application of data fusion based on clustering-neural network for ETC gantry flow capacity correction. CICTP.

[10] Wu Y., Tan H., Li Y., Zhang J., Chen X. (2018). A fused CP factorization method for incomplete tensors. IEEE Trans. Neural Netw. Learn. Syst..

[11] Chiou J.M., Zhang Y.C., Chen W.H., Chang C.W. (2014). A functional data approach to missing value imputation and outlier detection for traffic flow data. Transp. B Transp. Dyn..

[12] Li L., Li Y., Li Z. (2013). Efficient missing data imputing for traffic flow by considering temporal and spatial dependence. Transp. Res. Part C Emerg. Technol..

[13] Zhu Z., Xu M., Wang K., Lei C., Xia Y., Chen X. (2023). A non-local grouping tensor train decomposition model for travel demand analysis concerning categorical independent variables. Transp. Res. Part C Emerg. Technol..

[14] Chen X., He Z., Wang J. (2018). Spatial-temporal traffic speed patterns discovery and incomplete data recovery via SVD-combined tensor decomposition. Transp. Res. Part C Emerg. Technol..

[15] Chen X., He Z., Sun L. (2019). A Bayesian tensor decomposition approach for spatiotemporal traffic data imputation. Transp. Res. Part C Emerg. Technol..

[16] Li L., Zhang J., Wang Y., Ran B. (2018). Missing value imputation for traffic-related time series data based on a multi-view learning method. IEEE Trans. Intell. Transp. Syst..

[17] Zhang Y.F., Thorburn P.J., Xiang W., Fitch P. (2019). SSIM—A deep learning approach for recovering missing time series sensor data. IEEE Internet Things J..

[18] Chen Z., Lu Z., Chen Q., Zhong H., Zhang Y., Xue J., Wu C. (2022). A spatial-temporal short-term traffic flow prediction model based on a dynamical-learning graph convolution mechanism. arXiv.

[19] Han L., Zheng K., Zhao L., Wang X., Wen H. (2020). Content-aware traffic data completion in ITS based on generative adversarial nets. IEEE Trans. Veh. Technol..

[20] Yang B., Kang Y., Yuan Y., Huang X., Li H. (2021). ST-LBAGAN: Spatio-temporal learnable bidirectional attention generative adversarial networks for missing traffic data imputation. Knowl. Based Syst..

[21] Lu W., Zhou T., Li L., Gu Y., Rui Y., Ran B. (2022). An improved Tucker decomposition-based imputation method for recovering lane-level missing values in traffic data. IET Intell. Transp. Syst..

[22] Deng L., Liu X.Y., Zheng H., Feng X., Chen Y. (2021). Graph spectral regularized tensor completion for traffic data imputation. IEEE Trans. Intell. Transp. Syst..

[23] Wu H., Yang C., Xie W., Zhang W. (2021). Joint matrix decomposition-based missing data completion in low-voltage area. Math. Probl. Eng..

[24] Tan H., Feng G., Feng J., Wang W., Zhang Y.-J., Li F. (2013). A tensor-based method for missing traffic data completion. Transp. Res. Part C Emerg. Technol..

[25] Zhang X., Zhang Y., Wei X., Hu Y., Yin B. (2022). Traffic forecasting with missing data via low rank dynamic mode decomposition of tensor. IET Intell. Transp. Syst..

[26] Wang J., Wu J., Wang Z., Gao F., Xiong Z. (2020). Understanding urban dynamics via context-aware tensor factorization with neighboring regularization. IEEE Trans. Knowl. Data Eng..

[27] Dong H., Ding F., Tan H., Zhang H. (2022). Laplacian integration of graph convolutional network with tensor completion for traffic prediction with missing data in inter-city highway network. Phys. A Stat. Mech. Appl..

[28] Liu C., Wu T., Li Z., Wang B. (2021). Individual traffic prediction in cellular networks based on tensor completion. Int. J. Commun. Syst..

[29] Liu C., Wu T., Li Z., Ma T., Huang J. (2022). Robust online tensor completion for IoT streaming data recovery. IEEE Trans. Neural Netw. Learn. Syst..

[30] Li L., Lin X., Liu H., Lu W., Zhou B., Zhu J. (2022). Displacement Data Imputation in Urban Internet of Things System Based on Tucker Decomposition with L2 Regularization. IEEE Internet Things J..

[31] Gong C., Zhang Y. (2020). Urban traffic data imputation with detrending and tensor decomposition. IEEE Access.

[32] Qin J., Ma Q., Yu X., Wang L. (2020). On synchronization of dynamical systems over directed switching topologies: An algebraic and geometric perspective. IEEE Trans. Autom. Control.

[33] Cui Z., Henrickson K., Ke R., Wang Y. (2020). Traffic graph convolutional recurrent neural network: A deep learning framework for network-scale traffic learning and forecasting. IEEE Trans. Intell. Transp. Syst..

